# Genome-wide analysis of the *Saccharina japonica* sulfotransferase genes and their transcriptional profiles during whole developmental periods and under abiotic stresses

**DOI:** 10.1186/s12870-020-02422-3

**Published:** 2020-06-11

**Authors:** Chang Lu, Zhanru Shao, Pengyan Zhang, Delin Duan

**Affiliations:** 1 Key Laboratory of Experimental Marine Biology, Center for Ocean Mega-Science, Institute of Oceanology, Chinese Academy of Sciences, Qingdao, 266071 P. R. China; 2 Laboratory for Marine Biology and Biotechnology, Qingdao National Laboratory for Marine Science and Technology, Qingdao, 266237 P. R. China; 3 University of Chinese Academy of Sciences, Beijing, 100049 P. R. China; 4 Division of Mariculture Ecology and Technology, Yellow Sea Fisheries Research Institute, Chinese Academy of Fishery Sciences, Qingdao, 266071 China; 5 State Key Laboratory of Bioactive Seaweed Substances, Qingdao Bright Moon Seaweed Group Co Ltd, Qingdao, 266400 P. R. China

**Keywords:** *Saccharina japonica*, Sulfotransferase, Fucoidan, Transcriptome, Genome

## Abstract

**Background:**

As a unique sulfated polysaccharide, fucoidan is an important component of cell wall in brown seaweeds. Its biochemical properties are determined by the positions and quantity of sulfate groups. Sulfotransferases (STs) catalyze the sulfation process, which transfer the sulfuryl groups to carbohydrate backbones and are crucial for fucoidan biosynthesis. Nevertheless, the structures and functions of *STs* in brown seaweeds are rarely investigated.

**Results:**

There are a total of 44 *ST* genes identified from our genome and transcriptome analysis of *Saccharina japonica*, which were located in the 17 scaffolds and 11 contigs. The *S. japonica ST* genes have abundant introns and alternative splicing sites, and five tandem duplicated gene clusters were identified. Generally, the *ST* genes could be classified into five groups (Group I ~ V) based on phylogenetic analysis. Accordingly, the ST proteins, which were encoded by genes within the same group, contained similar conserved motifs. Members of the *S. japonica ST* gene family show various expression patterns in different tissues and developmental stages. Transcriptional profiles indicate that the transcriptional levels of more than half of the *ST* genes are higher in kelp basal blades than in distal blades. Except for *ST5* and *ST28,* most *ST* genes are down-regulated with the kelp development stages. The expression levels of nine *ST genes* were detected by real-time quantitative PCR, which demonstrates that they responded to low salinity and drought stresses.

**Conclusions:**

Various characteristics of the *STs* allow the feasibilities of *S. japonica* to synthesize fucoidans with different sulfate groups. This enables the kelp the potential to adapt to the costal environments and meet the needs of *S. japonica* growth.

## Background

*Saccharina japonica* is a brown seaweed with high commercial value in Asia. It is rich in crude fibers and carbohydrates and is widely used as a raw material for the extraction of alginate and fucoidan. Moreover, *S. japonica* contains many bioactive substances that are valuable for cosmetics, foods and health [1]. Among all bioactive metabolites, fucoidan, a sulfated polysaccharide, is considered highly valuable in the field of medicine. For instance, fucoidan exerts immunomodulation, anti-inflammation, anti-tumor, anticoagulant and antithrombotic functions [2–5], and is also effective in relieving diabetic nephropathy and adenine-induced chronic kidney disease [6, 7].

Fucoidan, which mainly exists in echinoderm and cell walls of brown algae [8], was first discovered by Kylin in brown algae *Laminaria digitata* in 1913 [9]. The fucoidan biosynthesis pathway in brown algae was not clear until the release of genome sequences of *Ectocarpus siliculosus* in 2010 [10]. Based on *E. siliculosus* genome sequencing and analogized with glycosaminoglycan (GAG) biosynthesis, Michel et al. (2010) deduced that fucoidan may first be polymerized into neutral polysaccharides by fucosyltransferases, and then sulfated by specific sulfotransferases [11]. He proposed two routes of GDP-fucose production: 1) fructose-6-phosphate is catalyzed by mannose-6-phosphate isomerase (MPI), phosphomannomutase (PMM) and mannose-1-phosphate guanylyltransferase (MPG) to synthesize GDP-mannose, followed by the production of GDP-fucose, which is catalyzed by GDP-mannose 4, 6-dehydrogenase (GM46D) and the bifunctional enzyme GDP-L-fucoidase synthase (GFS); 2) alternatively, L-fucose is used as the substrate to synthesize GDP-fucose by fucose kinase (FK) and GDP-fucose pyrophosphorylase (GFPP). GDP-fucose is subsequently used to generate fucoidan by fucosyltransferase (FUT) and sulfotransferase (ST). Some genes involved in fucoidan biosynthesis have been investigated in *S. japonica* and *Nemacystus decipiens* [12, 13]. Chi et al. (2017) explored the gene origin, expression difference and the enzymatic activity of MPI, MPG and PMM in *S. japonica* [14]*.* Nishitsuji et al. (2019) confirmed that *FK* and *GFPP* fused in *N. decipiens* genome [13]. Zhang et al. (2018) illustrated the expression and purification, enzymatic activity and response to light and temperature stress of PMM/PGM (phosphoglucomutase) in *S. japonica* [15].

There are many kinds of monosaccharide involved in the biosynthesis process of fucoidan. The main component of the sulfated fucoidan was L-fucose-4-sulfate; galactose, mannose, xylose, glucose, arabinose, and glucuronic acid exist in small amounts [16, 17]. It was believed that the content and structure of fucoidans in algae vary in different algae species, tissues, age, inhabitance and seasons [18, 19]. The structural parameters of fucoidan, such as the type of monosaccharide and fucose chain and the molecular weight of polysaccharide, all contribute to its bioactivity, especially the number and position of sulfate groups on the macromolecular skeleton [20–22]. For instance, the 2, 3-disulfated sugar residue is a common structure for anticoagulant activity [23, 24], whereas, the existence of 2-O-sulfation at the C-2 position reduces the anticoagulant activity of fucoidan [25]. Thus, sulfation has an influence on the function of fucoidan. It has been reported that sulfotransferase (ST) transfers the sulfuryl groups from the universal donor 3′-phosphoadenosine 5′-phosphosulfate (PAPS) to carbohydrate backbones [26]. Therefore, sulfotransferase, the crucial enzyme catalyzing the last step of fucoidan biosynthesis determines the position and quantity of sulfate groups in fucoidan. Multiple *ST* sequences have been annotated in genome of many kinds of algae, e.g. 41 in *E. siliculosus* and 24 in *Cladosiphon okamuranus* [10, 27]. Besides, Ye et al. (2015) reconstructed the carbon metabolism pathway in 14 algal genomes [12], and 13 out of 14 species genomes contains *ST* genes. Considering *ST* gene family contains large amount of putative members, it is thus necessary to globally analyze the distinct features of these *STs* in brown algae. However, there is no study on the *ST* gene family of brown algae so far.

In this study, we screened 104 genes (including three *MPIs*, two *PMMs*, three *GM46Ds*, 22 *FUT*, 73 *ST* and one *FK*) involved in fucoidan biosynthesis in *S. japonica*. Specifically, we characterized the *ST* genes by analyzing their sequence features, scaffold locations, phylogenetic relationships, tissue and time specific expression patterns and dynamic transcriptional profiles in response to low salinity and drought abiotic stresses. This is the first study to investigate the characteristics of *ST* family members in *S. japonica*. Our results provide valuable knowledge of the biosynthesis of sulfated fucoidan in brown seaweeds, and have great potential for in vitro applications of STs in fucoidan synthesis.

## Results

### Identification and expression profiles of fucoidan biosynthetic genes

**Fig. 1:**
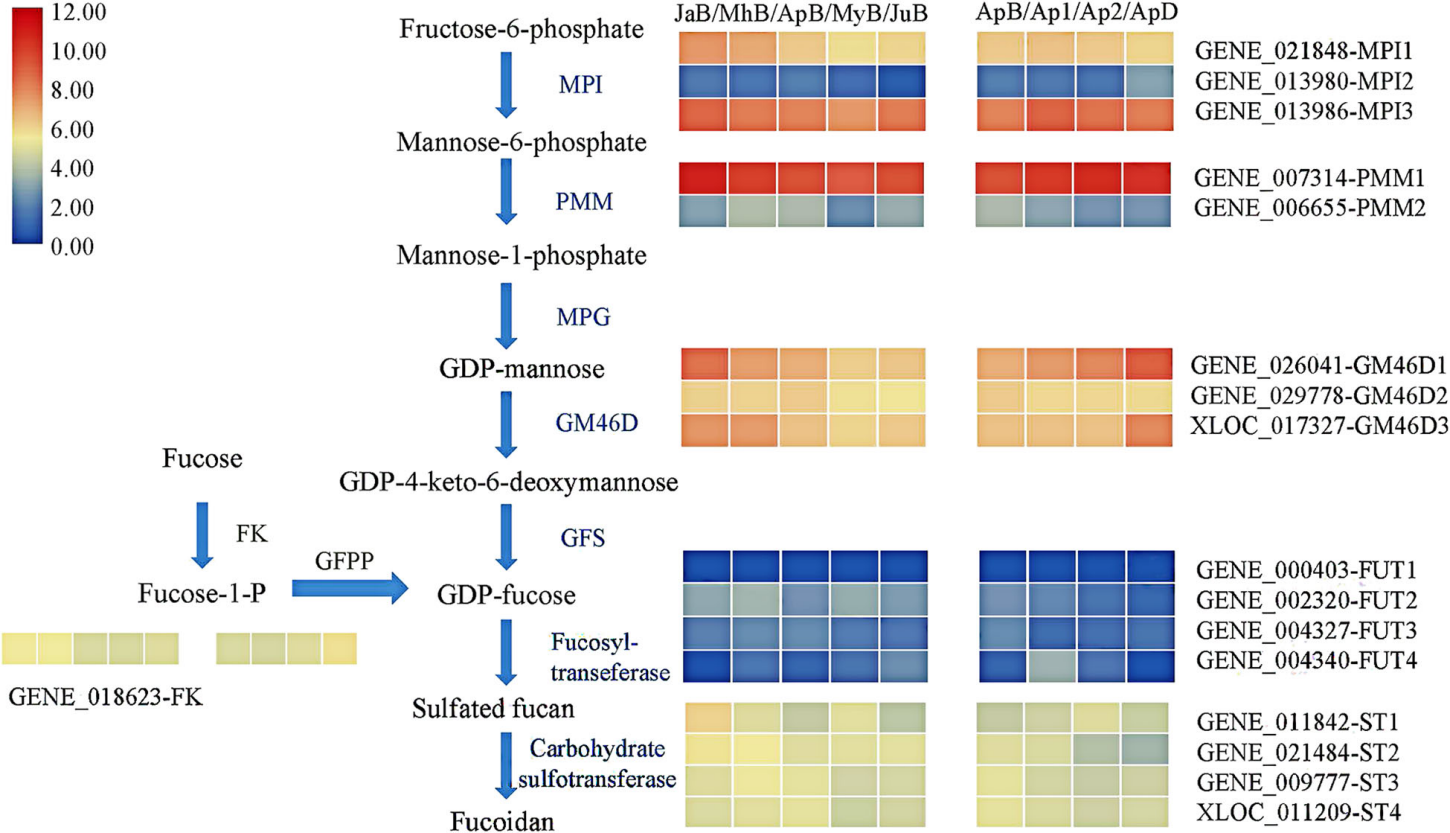
Fucoidan biosynthesis pathway in *S. japonica* and expression levels of representative genes. The order of each row is: Jan-basal, Mar-basal, Apr-basal, May-basal, Jun-basal. MPI: mannose-6-phosphate isomerase, PMM: phosphomannomutase, MPG: mannose-1-phosphate guanylyltransferase, GM46D: GDP-mannose 4, 6-dehydrogenase, GFS: GDP-L-fucoidase synthase, FK: fucose kinase, GFPP: GDP-fucose pyrophosphorylase

A total of 104 genes related to fucoidan biosynthesis were annotated based on our genome and transcriptome databases of *S. japonica*. Table S1 lists their gene ID, length of sequences and FPKM values (Additional file 1: Table S1). Figure 1 shows the expression levels of corresponding genes. The transcriptional levels of *MPI3* (GENE_013986), *PMM1* (GENE_007314), *GM46D1* (GENE_026041) and *ST1* (GENE_011842) were relatively higher in each catalytic step, which were believed to be essential for fucoidan biosynthesis during the kelp growth and development. From the perspective of expression abundance, *PMM1* showed the highest FPKM value while all the *FUTs* were expressed in very low levels. In different development stages, most of these genes were shown a down-regulated trend. In different tissues, *MPI2*, *PMM1*, *GM46D1*, *GM46D3* and *FK* showed up-regulated trends from basal blade to distal blade while *PMM2*, *GM46D2*, *FUT2*, *FUT3* were down-regulated.

### Identification and sequences characterization of the *ST* genes

*S. japonica* genome had 73 genes automatically annotated as *ST* genes. After further analysis with Blast, SMART and Pfam database, the sequences with low confidence of sulfotransferase domain and repetitive genes were removed. Finally, 44 sequences were confirmed as *S. japonica ST* genes, which at least contained one of these domains: *Gal-3-O_sulfotr* domain (PF06990), *Sulfotransfer_1* domain (PF00685), *Sulfotransfer-2* domain (PF03567) or *Sulfotransfer_3* domain (PF13469).

These 44 *ST* genes were named *ST1* to *ST44* (the average FPKM values from high to low). Name, gene ID, scaffold location, ORF length, exon number, amino acid number, molecular weight, and pI of the 44 genes and their corresponding proteins are summarized in Table 1. The number of amino acids of STs ranged from 82 (ST42) to 514 (ST38), and their molecular weight were from 9.57 kDa (ST42) to 56.82 kDa (ST38). The predicted isoelectric point (pI) values of ST proteins ranged from 4.66 (ST34) to 10.16 (ST29).

**Table 1:** Features of the *S. japonica ST* genes and their corresponding proteins

Gene	ID	Scaffolds	Genomic Location	ORF	Exon	AA	MW (kDa)	pIs
*ST1*	GENE_011842	chr4	10,762,476–10,778,806	1272	13	423	48.36	5.99
*ST2*	GENE_021484	chr27	754,976–771,068	1416	8	471	51.37	6.17
*ST3*	GENE_009777	chr15	5,230,475–5,248,182	1266	10	421	46.49	8.35
*ST4*	XLOC_011209	chr4	10,825,718–10,851,003	1266	12	421	48.31	5.13
*ST5*	GENE_026617	contig2134	6272–25,732	1236	9	411	46.61	9.40
*ST6*	GENE_013439	chr4	12,759,861–12,768,682	1134	5	377	40.53	5.14
*ST7*	GENE_011825	chr4	10,671,246–10,701,618	1281	13	426	48.49	5.99
*ST8*	GENE_026961	chr10	4,062,078–4,075,767	972	7	323	36.23	5.88
*ST9*	GENE_019245	contig569	94,071–106,934	783	8	260	28.80	9.26
*ST10*	XLOC_015241	chr3	8,411,455–8,434,996	1050	10	349	38.47	5.72
*ST11*	GENE_005471	chr19	3,980,992–4,011,984	1296	11	431	48.54	5.89
*ST12*	XLOC_024652	chr6	11,475,841–11,490,125	942	7	313	35.33	6.50
*ST13*	GENE_024538	contig148	34,484–58,037	1158	11	385	44.10	6.57
*ST14*	XLOC_026654	chr14	13,042,373–13,061,909	354	3	117	13.48	4.77
*ST15*	GENE_016486	chr29	6,287,160–6,299,378	822	9	273	30.81	5.96
*ST16*	GENE_018833	chr23	7,201,518–7,212,302	948	6	315	34.92	7.22
*ST17*	GENE_006499	chr6	1,127,751–1,135,830	600	6	199	22.72	4.87
*ST18*	GENE_017164	contig3228	10,008–34,404	1320	8	439	49.59	8.72
*ST19*	GENE_016121	chr25	1,006,090–1,010,442	1371	8	456	51.17	9.32
*ST20*	GENE_027595	chr3	8,856,825–8,882,319	1146	9	381	43.22	6.03
*ST21*	GENE_014314	contig3637	6130–10,174	1032	5	343	37.22	8.76
*ST22*	GENE_019315	contig793	48,109–52,653	828	6	275	31.70	5.84
*ST23*	GENE_018873	chr23	7,165,015–7,173,771	1233	8	410	45.28	6.29
*ST24*	XLOC_010290	contig1790	17,173–23,856	489	5	162	18.81	5.42
*ST25*	GENE_007603	chr16	3,011,123–3,025,060	1251	11	416	47.56	5.68
*ST26*	GENE_016729	chr14	16,108,770–16,114,283	1179	7	392	43.69	9.83
*ST27*	GENE_015508	chr9	15,532,393–15,534,802	867	3	288	22.62	6.59
*ST28*	GENE_019207	chr13	8,509,051–8,518,818	1254	5	417	46.00	9.19
*ST29*	GENE_015136	chr12	6,892,181–6,895,811	366	4	121	13.89	10.16
*ST30*	GENE_007786	contig317	372,098–396,742	1422	6	473	51.37	6.15
*ST31*	GENE_015734	contig88	64,588–75,181	984	8	327	34.56	6.15
*ST32*	XLOC_016866	chr14	13,173,117–13,177,606	861	6	286	32.02	6.56
*ST33*	GENE_000114	chr16	13,476,628–13,492,493	702	9	233	27.31	5.37
*ST34*	GENE_020104	contig1359	60,122–62,168	549	4	182	21.23	4.66
*ST35*	GENE_022140	chr19	7,743,111–7,755,418	1386	9	461	51.77	9.03
*ST36*	GENE_023232	chr29	4,968,806–4,971,862	339	2	112	12.68	6.74
*ST37*	GENE_023153	chr20	7,032,508–7,047,225	1194	6	397	41.70	6.10
*ST38*	GENE_019758	chr15	5,177,415–5,200,598	1545	12	514	56.82	9.19
*ST39*	GENE_017041	contig1066	203,894–217,026	1410	5	469	48.64	9.62
*ST40*	GENE_009569	chr14	16,256,503–16,263,748	555	2	184	20.32	6.08
*ST41*	GENE_011849	chr4	12,443,481–12,445,952	447	2	148	16.85	9.95
*ST42*	GENE_016416	chr30	1,868,233–1,868,544	249	1	82	9.57	7.73
*ST43*	GENE_020101	contig1359	23,708–25,725	603	4	200	23.70	5.44
*ST44*	GENE_026033	chr12	3,642,234–3,645,706	1062	3	353	38.47	9.22

The localization predication of ST proteins shows that they have complex cell compartmentalization (Additional file 2: Table S2). Prediction indicated that 70.45% proteins were non-secretory. Nine proteins (ST2, ST3, ST16, ST23, ST35, ST37, ST38, ST39 and ST41) have transmembrane helices, which may be located in plasma membrane or endomembrane system. Among them, four STs (ST3, ST16, ST37 and ST38) had signal peptides and two STs (ST2 and ST23) had signal anchors. In addition, seven proteins (ST1, ST4, ST6, ST7, ST10, ST11 and ST25) contained signal peptides without the transmembrane domain. ST2, ST13 and ST26 were predicted to target the chloroplast with high confidence by TargetP, whereas ST3, ST7, ST9, ST14, ST16, ST19, ST22, ST25, ST35, ST38, ST43 and ST44, were predicted to be located in the mitochondria.

### Phylogenetic, motif and gene structure analysis of the *ST* genes

**Fig. 2:**
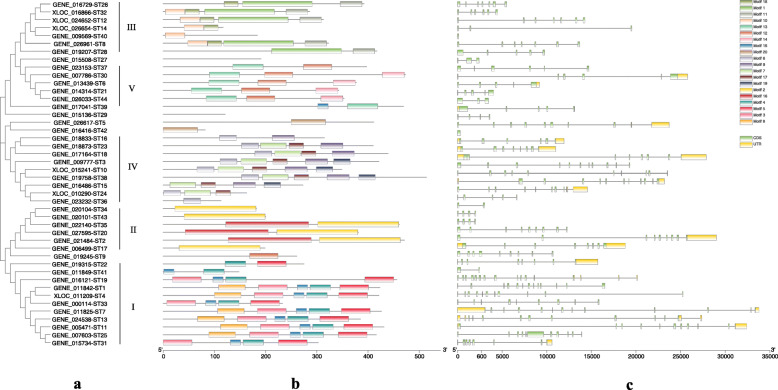
Phylogenetic relationship, conserved motifs and gene structure of the *ST* genes and their corresponding proteins in *S. japonica*. **a**: Phylogenetic tree of the 44 *S. japonica* ST proteins. The maximum likelihood phylogenetic tree was constructed using MEGA 7.0.26, with 1000 bootstrap replications, WAG+F + G method, Gamma 4, partial deletion and 50% site coverage as the cutoff. **b**: Conserved motifs identified in the 44 ST proteins. Twenty putative motifs were indicated by boxes of different color. Details on the motifs are listed in Additional file 3: Table S3. **c**: Structures of the *ST* genes. Exons, introns and UTRs are indicated by green boxes, black lines and yellow boxes, respectively

The 44 *S. japonica* ST proteins could be primarily classified into five groups (I - V) (Fig. 2a). A total of 20 conserved motifs were identified by MEME, which length ranged from 21 to 163 amino acids (Fig. 2b and Additional file 3: Table S3). The ST amino acid sequences in the same group contain similar conserved motifs and indicated that they were highly conserved (Fig. 2a and b). GroupI (25%) contained *Gal-3-O_sulfotr domain* (PF06990), group II (13.6%), III (15.9%) and V (11.36%) contained *Sulfotransfer_1* domain (PF00685) and *Sulfotransfer_3* domain (PF13469), group IV (20.45%) contained *Sulfotransfer-2* domain (PF03567). Motif 1, 16 and 20 were detected to have both *Sulfotransfer_1* domain (PF00685) and *Sulfotransfer_3* domain (PF13469). Besides, *Gal-3-O_sulfotr* and *Sulfotransfer_3* domain are only present in algae [28].

Gene structure analysis showed that *ST* gene family included multiple introns. The number of introns ranged from 0 to 12. Each *ST* contained 6.95 introns on average. Most *ST* genes (77.3%) had more than three introns. Only one gene (*ST42*) had no introns. The longest intron identified in *ST* genes was nearly 15 kb (Fig. 2c).

**Fig. 3:**
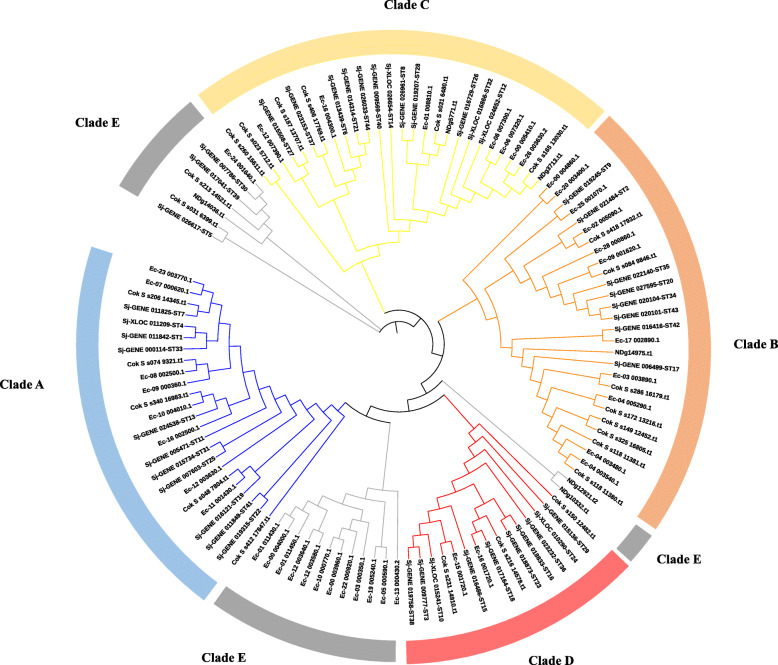
Phylogenetic tree of the ST proteins from *E. siliculosus*, *C. okamuranus*, *N. decipiens* and *S. japonica*. A maximum likelihood phylogenetic tree was constructed based on full-length amino acid sequences of the 115 STs, with 1000 bootstrap replications, the WAG+F + G model, Gamma 2, partial deletion and 50% site coverage as the cutoff. These 115 ST proteins were clustered into five subfamilies. Sequence were listed in Additional file 4: Table S4

To study the evolutionary relationship among *STs* annotated from *S. japonica* and other brown algae, a maximum likelihood (ML) phylogenetic tree was constructed based on the ST amino acid sequences: 44 from *S. japonica*, 41 from *E. siliculosus*, 24 from *C. okamuranus* and six from *N. decipiens* (Fig. 3 and Additional file 4: Table S4). Five ST clades were divided, including clade A (24), B (28), C (27), D (15) and E (21), respectively. The group III and V of 44 *ST* genes in *S. japonica* in Fig. 2 were divided into clade C and showed a closer evolutionary relationship than other groups. STs in the same clade contained same domains. ST5, ST30 and ST39 had far evolutionary distance with other STs in *S. japonica.* Each clade contained STs from *S. japonica, E. siliculosus* and *C. okamuranus.* Interestingly, 12 STs of *E. siliculosus* formed a group between clade A and clade D and contained *Sulfotransfer-2* domain (PF03567). STs from *N. decipiens* were only found in clade B, C and E.

### Alternative splicing analysis of *STs*

We analyzed the types and numbers of all alternative splicing (AS) sites in *S. japonica ST* genes in different tissues and developmental stages. A total of 217 sites were identified in this gene family. The most abundant AS site type was the alternative transcription start site (TSS) type (72), followed by exon skipping (ES, 52), other (31), p3_splice (21), p5_splice (19), alternative transcription terminal site (TTS, 13) and intergentic (9). Although types and number of AS sites were not uniform in different tissues and developmental stages, some genes centrally contained certain AS types, for example, *ST1* (p5_splice), *ST3* and *ST19* (p3_splice), *ST8* (TTS) and *ST9* and *ST26* (TSS). In the same sample, more AS sites were detected in *ST* genes with relatively high expression levels. In addition, more AS sites were discovered in basal blade samples than in distal blade. Details of these sites are listed in Additional file 5: Table S5.

### Scaffold location and gene duplication of the *STs*

**Fig. 4:**
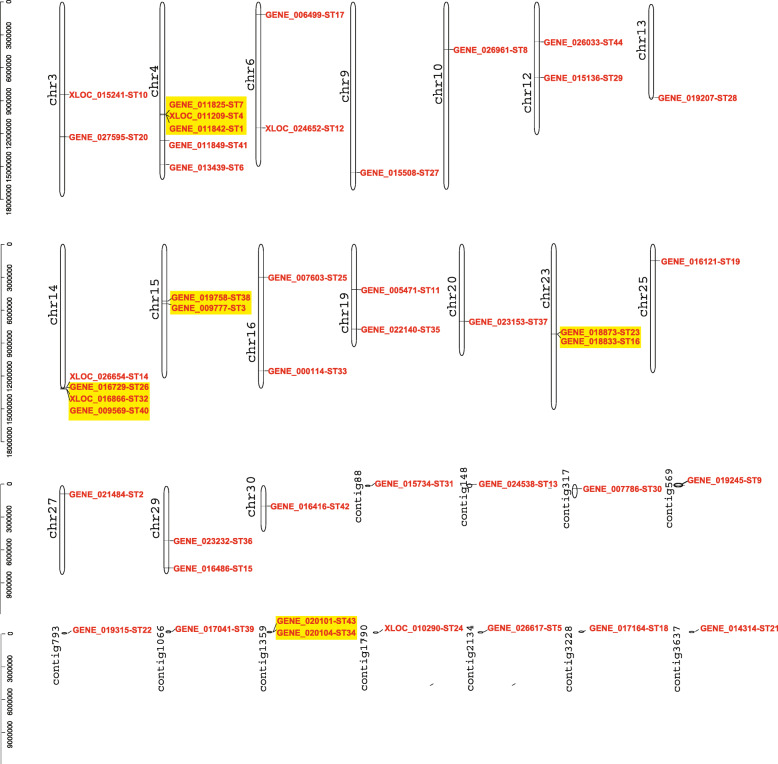
Scaffold locations of the *ST* genes and identification of duplicate genes. Tandem duplicated genes are indicated by yellow background

**Fig. 5:**
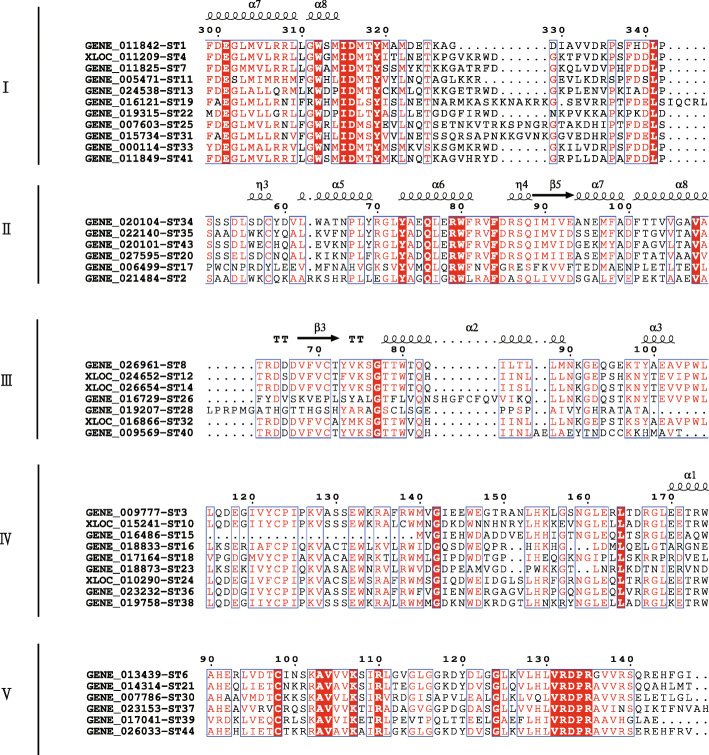
Representative secondary structures and sequence alignment of STs in each group. Amino acids in white on a red background are conserved sites and those in red with blue rectangles are similar. The secondary structure of STs is shown above the alignment. Alpha-helices are represented as helices symbols. β-strands with arrows and turns with TT letters

*ST* genes loci distributed randomly and dispersedly on 17 scaffolds and 11 contigs in *S. japonica* genome (Fig. 4). Scaffold 4 and 14 contained five and four *ST* loci, respectively. Although the generation of gene family was usually attributed to tandem duplication and segmental duplication [29], the *ST* family only contained tandem duplication and these five pairs of genes covered 27.3% of the whole *ST* gene family. Genes in the same tandem duplicated pair were located in the same scaffold or contig and demonstrated close physical distance. Usually, there is only one sequence on a chromosome, or two sequences distantly appeared on the same chromosome. Duplicated *ST* gene pairs were found on scaffold 15 (*ST3* and *ST38*), 23 (*ST16* and *ST23*) and contig1359 (*ST34* and *ST43*). Two groups of three tandem duplicated genes, *ST1*, *ST4* and *ST7* were identified on scaffold 4, and *ST14*, *ST26* and *ST32* were identified on scaffold 14. We found highly similar gene structure, conserved motif and protein secondary structure (Fig. 5) in the same pairs of tandem duplicated genes. No collinearity among *ST* family members was observed with MCScanX.

### Secondary structure analysis of ST proteins

The secondary structure of protein mainly refers to the main peptide chain curls and folds regularly with hydrogen bond to form a conformation with periodic structure along one-dimensional direction. We used SOPMA to predict the protein structure of STs and found four secondary structures, which randomly distributed in all peptide chain, including alpha-helix, extended strand, beta-turn and random coil (Additional file 6: Table S6). Alpha-helix and random coil were the major components of secondary structure and accounted for 41.91 and 39.87% on average, respectively. The proportion of extended strand was 12.58%. The least was beta-turn, only 5.65%. In five groups of Fig. 2, the proportion of alpha-helix of group III was the lowest, only 34.19%, while other four groups contained more than 40% alpha-helix. Genes in same tandem duplicated pairs (Fig. 4) showed similar proportions of these four secondary structures. Figure 5 shows the representative secondary structures of each group.

### Transcriptional profiles of *STs* in different tissues and developmental stages

**Fig. 6:**
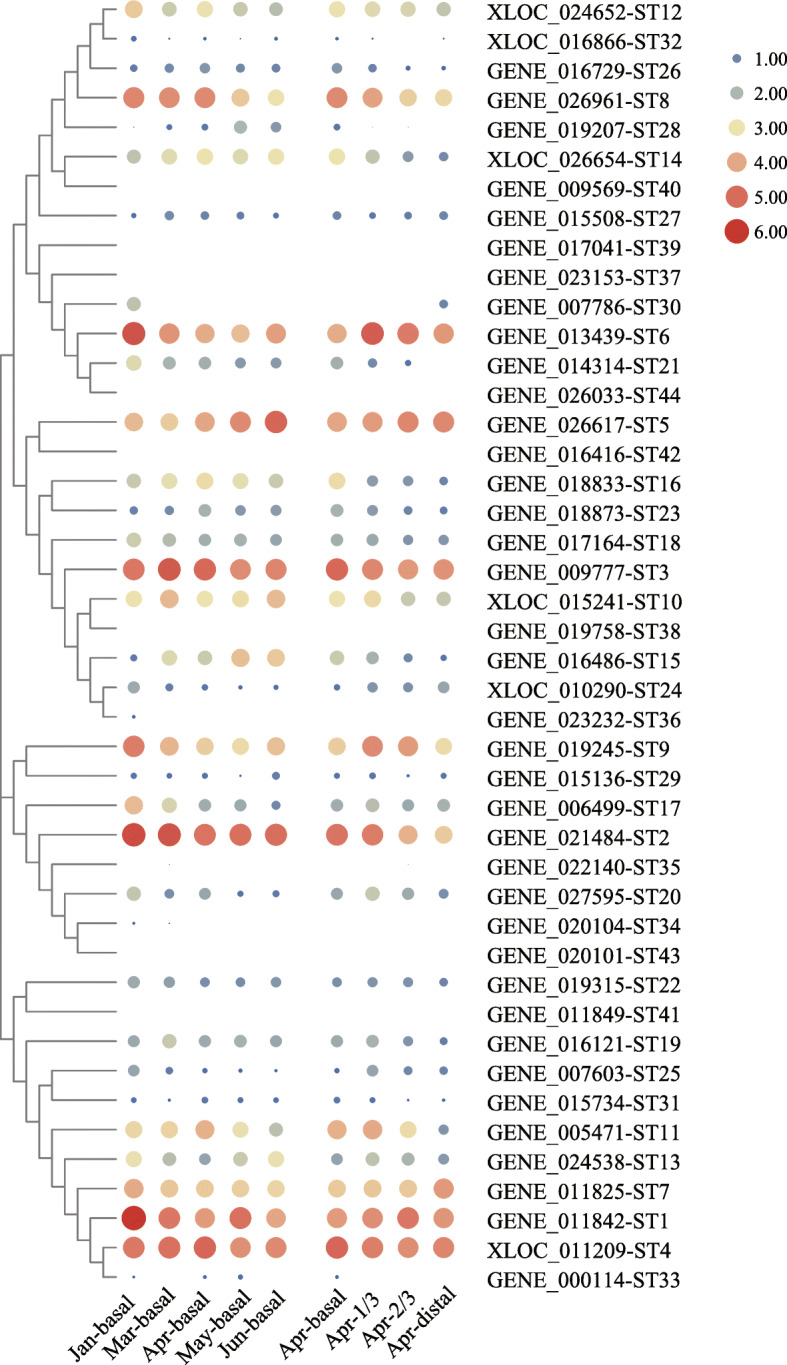
Transcript profiles of the *S. japonica ST* genes in different tissues and developmental stages. The larger the circle, the higher the gene expression. If the expression of one gene detected at a time point was 0, the corresponding position was blank. The heatmap was constructed by TBtools

Based on the RNA-Seq data, a heatmap of *ST* genes under various tissues and developmental stages was illustrated by TBtools (Fig. 6). In the tested samples, *ST1* to *ST36*, *ST38* and *ST39* were expressed at least in one tissues or developmental stage, and *ST1* to *ST10* were always highly expressed in all samples. In contrast, the FPKM values of *ST37* and *ST40* to *ST44* were zero all the time. In different developmental stages, *ST1*, *ST17* and *ST25* were obviously down-expressed, while *ST5*, *ST15* and *ST28* were markedly up-expressed. From basal to distal blade, *ST11*, *ST15* and *ST21* had a down-regulation trend, while *ST30* and *ST24* showed an up-regulation trend. More than half of the *STs* (54.5%) were more expressed in the basal than in the distal blade. The expression levels of *ST13*, *ST16* and *ST19* had no significant change. It is noteworthy that three out of five pairs of tandem duplicated genes (*ST1*, *4*, *7*; *ST14*, *26*, *32*; *ST16*, *23*) showed similar expression trend, except for *ST34*, *ST43* and *ST38* with the too low expression level to be detected.

### Validation of qualification of RNA-Seq

**Fig. 7:**
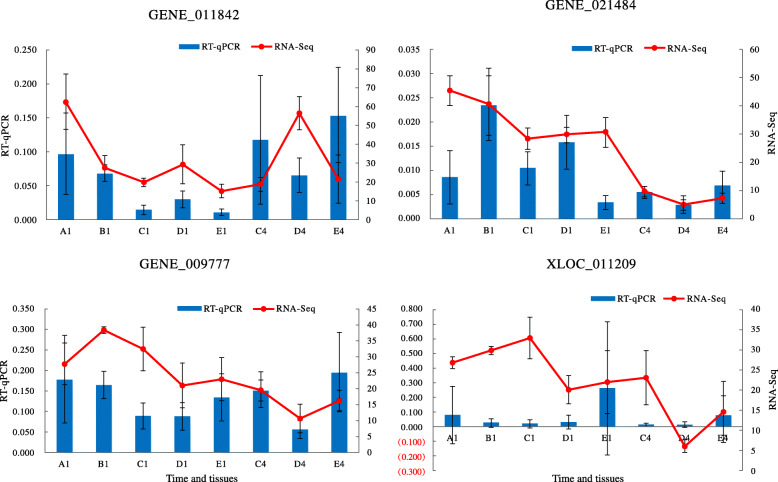
Expression profiles of the four selected *ST* genes. Quantitative RT-PCR was used to determine the transcript levels of four *STs*, GENE_011842, GENE_021484, GENE_009777 and XLOC_011209. Data are represented by mean ± standard deviation of two replicates. The relative transcript levels of the selected four *ST* genes were calculated using the 2^-ΔΔCt^ method with *β-actin* as the internal reference gene

We randomly selected three *ST* genes (GENE_011842, GENE_013439 and GENE_014314) for PCR amplification. The sequences of these three *STs* cloned from *S. japonica* cDNA templates showed that its genome and RNA-Seq databases were reliable (Additional file 7: Table S7). qRT-PCR was used to verify the transcript profiles of the four target *ST* genes with relatively high expression levels (GENE_011842, GENE_021484, GENE_009777 and XLOC_011209) involved in fucoidan biosynthesis. The qRT-PCR results and those of RNA-Seq were also consistent (Fig. 7). These two results indicated the reliability of our genome assembly and RNA-Seq data.

### Trend analysis and functional enrichment of DEGs

The trends of transcriptional levels of the *ST* genes in *S. japonica* basal blade from January to June are shown in Table 2. The *STs* exhibited several major expressed patterns: profile0 (*ST8*, *ST17*, *ST18*, *ST21* and *ST25*), profile2 (*ST6*, *ST24* and *ST32*), profile3 (*ST1*, *ST9* and *ST30*), profile6 (*ST12* and *ST20)*, profile25 (*ST11*), profile28 (*ST23*) and profile29 (*ST5* and *ST28*) (Table 2). Profile 0 and 29 are the two representative profiles, the former contains genes with a down-regulated expression pattern from January to June while the latter had an up-regulated trend. The most enriched pathways in profile 0 included photosynthesis, carbon fixation and metabolic pathway. Genes involved in ribosome, nitrogen metabolism, sulfur metabolism and inositol phosphate metabolism were enriched in profile 29 (Additional file 8: Table S8).

**Table 2:** Transcript profiles of the *S. japonica ST* genes during different development stages

Gene ID	Profile	Details
GENE_026961-ST8	Profile 0	
GENE_006499-ST17		
GENE_017164-ST18		
GENE_014314-ST21		
GENE_007603-ST25		
GENE_013439-ST6	Profile 2	
XLOC_010290-ST24		
XLOC_016866-ST32		
GENE_011842-ST1	Profile3	
GENE_019245-ST9		
GENE_007786-ST30		
XLOC_024652-ST12	Profile6	
GENE_027595-ST20		
GENE_005471-ST11	Profile25	
GENE_018873-ST23	Profile28	
GENE_026617-ST5	Profile29	
GENE_019207-ST28		
GENE_024538-ST13	Profile 1	
GENE_000114-ST33	Profile 8	

We then analyzed the transcriptional levels of the *STs* in different tissues: distal blade, 1/3, 2/3 and basal blade of *S. japonica* collected in April (Table 3). According to our results, the *STs* exhibited five major expression patterns: profile0 (*ST14*, *ST15* and *ST23*), profile1 (*ST8* and *ST26*), profile 3 (*ST28*), profile4 (*ST16*), profile9 (*ST2*, *ST11*, *ST19* and *ST21*) and profile17 (*ST20*). The transcriptional levels of most *STs* were decreased, as observed for profiles 0, 1, 4 and 9, and genes related to basal metabolism and photosynthesis were enriched in these profiles (Additional file 9: Table S9).

**Table 3:** Transcript profiles of the *ST* genes in different *S. japonica* tissues

Gene ID	Profile	Details
XLOC_026654-ST14	Profile 0	
GENE_016486-ST15		
GENE_018873-ST23		
GENE_026961-ST8	Profile 1	
GENE_016729-ST26		
GENE_019207-ST28	Profile 3	
GENE_018833-ST16	Profile 4	
GENE_021484-ST2	Profile 9	
GENE_005471-ST11		
GENE_016121-ST19		
GENE_014314-ST21		
GENE_027595-ST20	Profile 17	
GENE_015734-ST31	Profile 10	
GENE_007786-ST30	Profile 13	
GENE_013439-ST6	Profile 20	
GENE_019245-ST9	Profile 21	
GENE_007603-ST25		

### Expression profiles of *ST* genes under abiotic stress

**Fig. 8:**
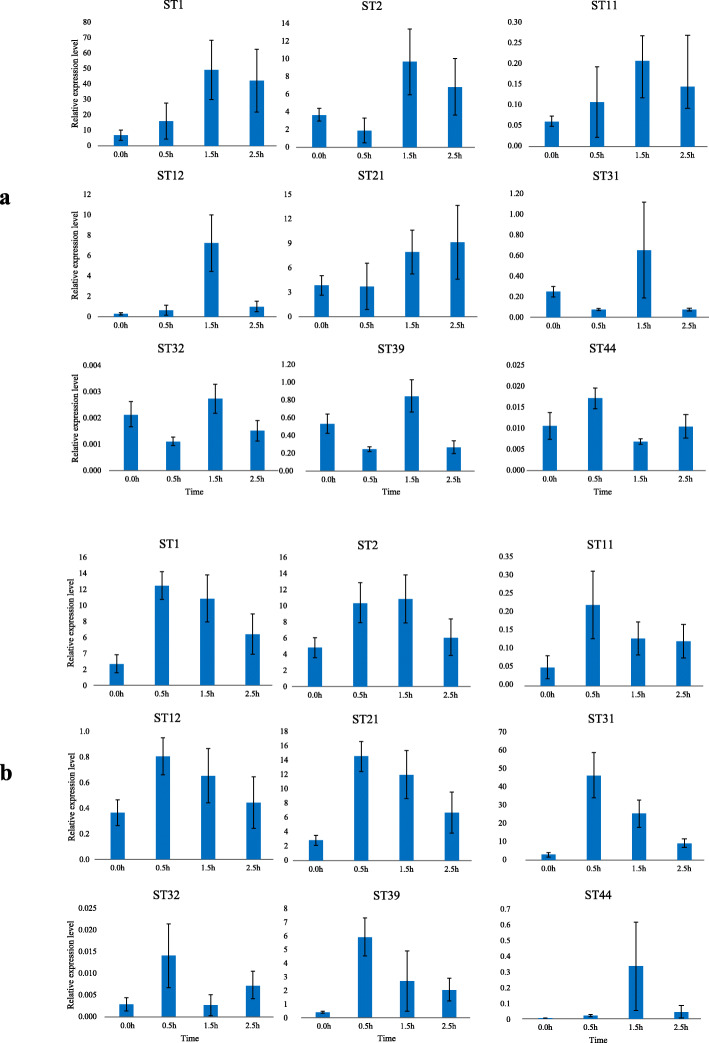
Relative expression levels of the nine selected *ST* genes. **a**: Expression profiles of *ST* genes under low salinity stress. **b**: Expression profiles of *ST* genes under drought stress. Data are represented by mean ± standard deviation of two replicates. The relative transcript levels of selected nine *ST* genes were calculated using the 2^-ΔΔCt^ method with *β-actin* as the internal reference gene

We used qRT-PCR to explore the variations of *STs* expression levels under low salinity and drought stresses. As shown in Fig. 8, *ST* genes were observed to be up-regulated under low salinity and drought conditions (Fig. 8a and b). These genes reached peak of expression at different treated time points. Under low salinity condition, *ST44* had the highest expression level at 0.5 h; *ST1*, *ST2*, *ST11*, *ST12*, *ST31*, *ST32* and *ST39* at 1.5 h; and *ST21* at 2.5 h. Additionally, the expression levels of *ST1*, *ST11*, *ST12* and *ST31* were up-regulated by more than four-fold at 1.5 h. *ST12* was the most up-regulated gene, and its expression level was 30-fold higher than that in the control group at 1.5 h. After drought treatment, the expression of all selected *STs* was significantly up-regulated. Most *STs* showed the highest expression level at 0.5 h. *ST2* and *ST44* reached peak at 1.5 h. The expression levels of *ST21*, *ST31*, *ST39* and *ST44* increased by more than four-fold at 0.5 h. As the most significant up-regulated gene, *ST44* was 67-fold as high as control group at 1.5 h. Under low salinity stress, the expression levels of most *ST* genes reached peak at 1.5 h and *ST* genes with high expression levels under normal condition were higher expressed. However, under drought stress, the highest *STs* expression levels mainly appeared at 0.5 h and *STs* with low expression levels in normal showed higher expression levels. The results showed that *ST* was more positively response to drought than low salinity stress.

## Discussion

In brown algae, sulfotransferase (ST) catalyzes the sulfation reaction in fucoidan biosynthesis, which determines the number and position of sulfate groups and is thus responsible for the various bioactivities of fucoidan. In this study, we retrieved 44 *ST* genes by screening the *S. japonica* genome and transcriptome databases and analyzed their structure, scaffold locations, phylogeny, duplication patterns and expression profiles in different tissues, developmental stages and under abiotic stresses. This study provides valuable information of the *ST* gene family and facilitates future studies on their functional divergence in brown algae.

### Multiple introns and AS may contribute to the diversity functions of STs

In eukaryotes, the process of AS is differentially spliced primary transcripts of many genes to produce multiple mRNAs. By selectively preserving or removing some exons, a single gene can be transcribed to produce a variety of proteins [30]. Basically, alternative splicing contributed to increased transcriptome and proteome diversity, and genes from most functional categories had high levels of AS [31]. Alternative splicing of intron is considered to regulate gene expression in different time and space [32]. Compared with *ST* families of *Arabidopsis* and *Oryza sativa*, which hardly contained introns [33, 34], the number of introns in *S. japonica ST* gene family was unexpectedly high. Combined with AS that found in this family, we presumed that the occurrence of introns and AS in *STs* could endow the cells synthesize a variety of ST enzymes with different functions and localization. This theory has been found in previous studies of other proteins. For example, it had been found that two alternatively spliced isoforms of serine-arginine-rich proteins in *Arabidopsis thaliana*, which were generated by 3′_splice, had distinct biological functions in plant development [35]. In plants and animals, the frequencies of AS types were decided by differences in their pre-mRNA splicing [36]. The organisms which contain large introns usually use an exon definition mechanism that results in exon skipping [37]. We correspondingly detected about 24% exon skipping (ES) sites in *ST* gene family and no intron retention (IR). Therefore, the type and quantity of the AS in *STs* are affected by the number of introns to some extent. We suggest that the production of multiple introns and AS is a developmental and physiological strategy which gradually formed in the evolution process of *S. japonica* for effective transcription.

### Tandem duplication has important sense in the expansion of *ST* gene family

Tandem duplication usually occurs in the region of chromosome recombination, forming a cluster of homologous genes with similar sequence and function, which arrange on the chromosome in a way of head and tail tandem. As a result, the number of one chromosome increases and the other decrease. This mechanism plays an indispensable role in the emergence of clustered genes [38]. Consistent with this conclusion, all the detected tandem duplicated *ST* genes appear in cluster, while other *ST* genes tend to appear alone. Tandem duplication tend to amplify dose insensitive genes and genes at the top or end of the metabolic pathway [39], which are also closely related to the amplification of genes related to biotic and abiotic stresses [40]. For example, Teng et al. (2017) found tandem duplication event of lipoxygenase gene family in *S. japonica* [41]. Considering the above theories, tandem duplication events may be significant in *S. japonica ST* gene family expansion.

### The members of *ST* gene family may have different protein structures and functions

Another observation is that similar motif organization only occurred in the same evolutionary subgroup of *S. japonica ST* genes. STs with highly similar motif distribution might produce similar three-dimensional structure and exercise similar functions. The different distribution of motifs between subgroups may further illustrate the various functions of *ST* genes.

From the phylogenetic tree of 115 STs, we observed that STs from different brown seaweeds could be found in each clade. This is consistent with their closer evolutionary relationships. Ye et al. (2015) had a similar report on the study of vBPO gene family [12]. Clade E contained the least STs from *S. japonica*, which may suggest the existence of gene loss evens in *S. japonica ST* gene family [42]. Twelve STs in *E. siliculosus* and two STs in *N. decipiens* clustered and formed two independent subgroups, which may due to their special functions or more independent evolutionary relationship.

### Different expression patterns of *STs* might meet the needs of growth and development of *S. japonica* in different environments

The expression levels of *STs* varied obviously in various developmental stages and tissues. Considering the monthly changes in the content of sulphate of fucoidan [43], multiple expression patterns may indicate the synthesis of fucoidan with different sulfated degree. As the concentration of sulfated polysaccharide and its sulphate degree had a positive correlation with salinity in halophytic species [44], we thought this change may be meaningful for *S. japonica* adapting coastal environment.

Previously, it was hypothesized that sulfotransferase-mediated sulfation affects the bioactivity of certain compounds, thereby modulating physiological processes to adapt to abiotic stresses [45]. Teng et al. (2017) found that the expression of a *ST* gene of *E. siliculosus* was up-regulated under low salinity stress condition [46]. In our study, the up-regulated *ST* genes (*ST1*, *2*, *11*, *12*, *21*, *31*, *32*, *39*, *44*) under abiotic stresses illustrated that they are a kind of stress resistance gene in *S. japonica*. In brown algae, fucose-containing sulfated polysaccharide not only activates as cell walls matrix, but also may have a significant role in coping with osmotic stress [14, 47]. Combined the above theories and RNA-Seq and qRT-PCR results, we inferred that a part of *ST* genes (e.g. *ST1*, *ST2*, *ST3*) are highly expressed in *S. japonica* grown at normal condition, which are necessary to maintain the basic needs of growth and development of *S. japonica*. Meanwhile, the other members (*ST39* and *ST44*) are remarkably up-regulated to synthesize fucoidans with high-degree sulfation in response to abiotic stresses, although they exhibit very low expression levels under normal conditions. Therefore, these genes with strong response can be used as the key candidate genes for further functional study on abiotic stress resistance. *STs* reached the peak expression levels at different treated time. This phenomenon implies that different *STs* had different response and regulation mechanisms. Various expression patterns are beneficial for *S. japonica* to maintain osmotic pressure stability in response to low salt stress and to keep algae moist in case of drought. Therefore, *ST* genes family is of great significance for *S. japonica* to adapt to the complex and changeable marine environment.

## Conclusions

A total of 44 *ST* genes which can be divided into five subgroups were identified through analyzing the genome and transcriptome database of *S. japonica*. Subsequently, these genes were analyzed from gene structure, phylogeny, scaffold location, secondary structures, gene duplication, alternative splices and expression patterns in different tissues, periods and under abiotic stresses. The alternative splice events and introns make the formation of more ST with different function and location become possible. The variable expression patterns of ST genes may contribute to the monthly-changed degree of sulfation of fucoidan and be significant for *S. japonica* adapting coastal environment. Also as a kind of stress resistance gene, the existence of *ST* genes is important for *S. japonica* to adapt to changeable marine habitats during whole developmental periods. Our report will be important to future functional verification for the *ST* genes and potential biochemical manipulation to fucoidan in vitro in the future.

## Methods

### Algal sample collection and treatment

*S. japonica* sporophytes were collected in December 8th, 2019 from cultivated rafts in Gaolv Aquatic Company in Rongcheng, Shandong, China. All robust samples of similar size were treated overnight in 10 °C incubator without light. For low salinity stress, sporophytes were cultured in 16 ‰ salinity seawater for 0 h, 0.5 h, 1.5 h and 2.5 h. For drought stress, sporophytes were exposed in air for 0 h, 0.5 h, 1.5 h and 2.5 h. Three robust individuals were set as biological repeats for each time point, rinsed with filtered seawater several times. Each sectioned tissue samples was snap frozen in liquid nitrogen, and stored at − 80 °C until total RNA isolation.

### Retrieval of fucoidan biosynthetic pathway in *S. japonica*

We have finished RNA-Seq of *S. japonica* sporophytes during whole developmental periods [48]. Based on this transcriptome data (NCBI: PRJNA512328) and our previously sequenced *S. japonica* genome (NCBI: MEHQ00000000), we identified 104 genes in fucoidan biosynthetic pathway.

### Identification of sulfotransferase family members in *S. japonica* genome

We searched key word “sulfortransferase” in transcriptome sequences annotation file. As long as the gene annotation result contained “sulfortransferase”, this gene can be selected as candidate genes for further identification. In this way, we identified 73 genes automatically annotated as *sulfotransferase* (*ST*) genes that catalyze the last step of fucoidan biosynthesis. Firstly, these 73 genes were submitted to local Blast to remove redundant genes. If the result of two nucleotide sequences alignment is more than 99% identity, we regarded these two as repeated sequence, and only keep the longer sequence. Secondly, the rest of genes were submitted to SMART (http://smart.embl-heidelberg.de/) [49, 50] and Pfam (http://pfam.xfam.org/search) [51] to confirm the presence of the conserved domain with cut-off scorn that E-value < 0.05, and only genes with *sulfotransferase* domains were retained. Finally, we ranked and renamed the rest 44 genes according to the monthly average genes expression from high to low.

### Sequence analysis, chromosomal localization and gene duplication

The open reading frames (ORFs) of high-confidence *ST* genes were predicted using ORF finder (https://www.ncbi.nlm.nih.gov/orffinder/). If a sequence was detected more than one ORF, we chose the longest one by default. The ExPASy ProtParam tool (https://web.expasy.org/protparam/) was used to analyze the physical and chemical properties of the deduced ST proteins, including molecular weight (MW), and amino acid (AA) composition. The SignalP-5.0 Server (http://www.cbs.dtu.dk/services/SignalP/) was used to predict signal peptides [52], and TMHMM (v2.0; http://www.cbs.dtu.dk/services/TMHMM/) was employed for predicting the transmembrane helices in the proteins. Possible localization to the chloroplast, mitochondrion and cytoplasm was predicted by Target-P (http://www.cbs.dtu.dk/services/TargetP/) [53].

Structure and conserved motifs of the *ST* genes was analyzed according to Zhao et al. [42]. The chromosomal positions of the *ST* genes were acquired by aligning the full-length *ST* nucleic acid sequences to the *S. japonica* genome. TBtools was used to display the chromosomal positions of *STs* and their relative physical distances [54].

We used MCScanX to search for duplicate genes in the *ST* family [55]. All the protein sequences of the coding genes in the kelp genome were compared in pairs. The comparison results were used as the files input to MCScanX software to predict the duplicated genes. The software selected the default standards, which were divided into singleton, tandem, proximal and dispersed duplication and the results were output [56, 57].

### Identification of alternative splicing events

Tophat 2.1.1 was used to analyze alternative splicing events in the 44 *ST* genes from RNA-Seq data [58]. We referred to the analysis and classification process of He et al. [59].

### Sequence alignment and phylogenetic analysis

All the 44 *S. japonica* ST proteins were aligned by MAFFT [60] with the default parameters and showed secondary structures by ESPript 3.0 [61]. To analyze the evolutionary relationships among the 44 STs in *S. japonica*, a maximum likelihood (ML) phylogenetic tree was constructed based on the full length amino acid sequences with MEGA 7.0.26 using the WAG+G + F model with 1000 bootstrap replications, Gamma 4, partial deletion and 50% site coverage as the cutoff value [62].

The amino acid sequences of STs derived from *E. siliculosus* (41), *C. okamuranus* (24), *N. decipiens* (6) and *S. japonica* (44) were subjected to phylogenetic analysis. Details of total 115 sequences are displayed in Additional file 4: Table S4. The maximum likelihood (ML) phylogenetic tree was constructed by MEGA 7.0.26 using the full-length amino acid sequences of 115 ST proteins with 1000 bootstrap replications, the WAG+F + G model, Gamma 2, partial deletion and 50% site coverage as the cutoff value [62]. By running the program “Find Best DNA / Protein Models” of MEGA 7.0.26, we analyzed and got the best building model and related parameters, namely, the WAG+F + G model we used to construct phylogenetic tree.

### Transcript profiling of the *ST* genes in different tissues and developmental stages

Differentially expressed genes (DEGs) across samples were identified according to Shao et al. [48]. The transcriptional profiles of the *S. japonica ST* genes in different tissues and developmental stages were determined, obtained, normalized and clustered [48]. The heatmap of *ST* gene expression was drawn by TBtools [54].

### Total RNA extraction and cDNA synthesis

Total RNA was extracted using a SPARKeasy Polysaccharide polyphenols/complex plant RNA kit (SparkJade Science Co., Ltd., China). The extracted RNA was quantified using a Nanodrop 2000 Spectrophotometer (Thermo Scientific, USA). First-strand cDNA was synthesized using a SPARKscript II RT Plus Kit (With gDNA Eraser) (SparkJade Science Co., Ltd., China) and stored at − 20 °C for subsequent analysis. All manipulations were operated following the manufacturers’ instructions.

### PCR amplification and sequencing of the *ST* genes

We randomly selected three genes (GENE_011842, GENE_013439 and GENE_014314) for PCR amplification. Primers used to amplify the full-length cDNA sequences of these three genes are listed in Table 4. PCR amplification was performed using the synthesized cDNA as the template. The 20 μL reaction mixture contained 10 μL 2 × Phanta Max Master Mix (Vazyme, China), 3 μL template, 1 μL of each of the forward and reverse primer (10 μM) and 5 μL ddH_2_O. The reaction mixtures were briefly centrifuged and placed in a thermal cycler (Takara, Japan). The conditions used for PCR were as follows: 95 °C for 5 min, followed by five cycles of 95 °C for 15 s, 65 °C for 15 s, 72 °C for 90 s, 30 cycles of 95 °C for 15 s, 60 °C for 15 s, 72 °C for 90 s, and a final extension at 72 °C for 10 min. The PCR products were purified using the gel-cutting recovery kit (Insight, China) and inserted into TOPO cloning vector using a 5 min TA/Blunt-Zero Cloning Kit (Vazyme, China). The 5 μL ligation mixture contained 1 μL 5 × TA/Blunt-Zero Cloning Mix and 4 μL purified PCR products (40 ng/μL). The mixture was briefly centrifuged and incubated at 37 °C for 10 min.

**Table 4:** Primers used for gene cloning and qRT-PCR

Reaction	Gene ID	Forward primer (5′-3′)	Reverse primer (5′-3′)
Genes cloning	GENE_011842	ATGGCACGACTCTCTCTCAG	TCAGAACTGGTTCATCGGCGGT
	GENE_013439	ATGTACTGTGTGTATGGCCT	CTACGGTTCATAACCTAGAGCATCC
	GENE_014314	ATGGTGCATGGCTTGGAGTG	CTATAGCTCATAACCTAGAG
qRT-PCR	GENE_011842	GAAACAAAACGGGGTGGACG	GGTCCGTGGTTGCTACTGAA
	GENE_021484	GCTGATAGTGGTGGACTCGG	CATGTCGTTGTGGTCGGAGA
	GENE_005471	ACAAGACCGGATCGACAACC	ACTTCTGGCTCTCTTGCGTC
	XLOC_024652	CAAGGGAAGGTGCAACAACG	AGATGTTCGCCTTCGGGTTT
	GENE_014314	TGAAGTCCATTCGCCTCACC	TGTTGTCAGCTTTGACCCGA
	GENE_015734	GCACTATCACATCGGCACCT	GGCTCTCGGAAGATGGTGAC
	XLOC_016866	GCGATTTCGAGACCAGGGAT	GTAGCTCTTGGTGCTCGGTT
	GENE_017041	GCTGACGACGGAGGAGTTAG	CCGTGCAATCCTCAGCCATA
	GENE_026033	CACCACACCAGAGCATCCTT	CGCAGCCTTGTAATCGAAGC
	GENE_009777	GCAATCTTGCTTCTGCGACC	CAATCTCAACACCAACGCCC
	XLOC_011209	TACCTGGCGAAACCACGAAA	TTCATCAAACCGCTCCGTCA

For transformation, 5 μL of each recombinant plasmids was mixed with 50 μL Trans1-T1 Phage Resistant Chemically Competent Cell (TRANS, China). The mixture was processed under manufacturers operating manual. Colony PCR was carried out using M13 primers and plasmids were sanger sequenced (Sangon, Shanghai, China). The coding sequences of the three *STs* are provided in Additional file 7: Table S7.

### Verification of target genes by qRT-PCR

qRT-PCR was used to validate qualification of RNA-seq for four genes (*ST1*, *ST2*, *ST3* and *ST4*) and detected the transcript levels of the nine target genes with different FPKM values (*ST1*, *ST2*, *ST11*, *ST12*, *ST21*, *ST31*, *ST32*, *ST39* and *ST44*) under low salinity and drought stresses. Gene-specific primers used for qRT-PCR were designed using Primer-BLAST (https://www.ncbi.nlm.nih.gov/tools/primer-blast/) (Table 4).

qRT-PCR was performed on a Takara Thermal Cycle Dice™ Real Time System (Takara, Japan). A 10 μL qRT-PCR reaction contained 5 μL 2 × SPARKscript II RT Plus Master Mix (SparkJade Science Co., Ltd., China), 1 μL template, 0.2 μL of each of the forward and reverse primers (10 μM), and 3.6 μL ddH_2_O. Conditions used for qRT-PCR were as follows: 95 °C for 2 min 30 s, followed by 40 cycles of 95 °C for 10 s and 60 °C for 30 s; and one cycle of 95 °C for 15 s, 60 °C for 60 s and 72 °C for 15 s. Three biological repeats and two technical replicates were performed. The relative transcriptional levels of the genes were calculated by the 2^-ΔΔCt^ method [63], and *β-actin* was used as the internal reference [64].

## Supplementary information

**Additional file 1: Table S1.** Genes involved in the biosynthesis of fucoidan in *S. japonica.*

**Additional file 2: Table S2.** Subcellular localization prediction of the ST proteins.

**Additional file 3: Table S3.** The putative motifs of ST proteins.

**Additional file 4: Table S4.** ST amino acids sequences from all the selected species for phylogenetic analysis.

**Additional file 5: Table S5.** The types of alternative splicing sites in *S. japonica ST* genes of all samples.

**Additional file 6: Table S6.** The proportion of secondary structures of STs in *S. japonica*.

**Additional file 7: Table S7.** Sequenced results of cloned and sequenced genes.

**Additional file 8: Table S8.** Transcriptional profiles enriched pathway in *S. japonica* different development stages.

**Additional file 9: Table S9.** Transcriptional profiles enriched pathway in *S. japonica* different tissues.

## Data Availability

The reference genome of *S. japonica* was released in GenBank at the National Centre for Biotechnology Information (NCBI) with the accession number of MEHQ0000000 (https://www.ncbi.nlm.nih.gov/nuccore/MEHQ00000000.1/). RNA-Seq data was deposited in the NCBI Sequence Read Archive (SRA) with accession number of PRJNA512328 (https://www.ncbi.nlm.nih.gov/bioproject/PRJNA512328). All of the datasets supporting the results of this article are included within the article and its Additional files. *ST* sequences of *E. siliculosus* were download from (https://bioinformatics.psb.ugent.be/orcae/overview/Ectsi). *ST* sequences of *N. decipiens and C. okamuranus* were download from (https://marinegenomics.oist.jp/gallery/).

## Abbreviations

AA: amino acid
DEGs: Differentially expressed genes
ES: Exon skipping
FK: Fucose kinase
FPKM: Fragments per kilobase of transcript per million mapped reads
FUT: Fucosyltransferase
GFPP: GDP-fucose pyrophosphorylase
GFS: GDP-L-fucoidase synthase
GM46D: GDP-mannose 4, 6-dehydrogenase
IR: Intron retention
ML: Maximum likelihood
MPG: Mannose-1-phosphate guanylyltransferase
MPI: Mannose-6-phosphate isomerase
MW: Molecular weight
NCBI: National Center for Biotechnology Information
NJ: Neighbor-joining
ORFs: Open reading frames
PAPS: 3′-phosphoadenosine 5′-phosphosulfate
PMM: Phosphomannomutase
qRT-PCR: Real-time quantitative polymerase chain reaction
ST: Sulfotransferase
TSS: Alternative transcription start site
TTS: Alternative transcription terminal site
UTR: Untranslated Region
